# Validating a Xylose Regulator to Increase Polyhydroxybutyrate Production for Utilizing Mixed Sugars from Lignocellulosic Biomass Using *Escherichia coli*

**DOI:** 10.4014/jmb.2306.06006

**Published:** 2023-09-18

**Authors:** Suk-Jin Oh, Hong-Ju Lee, Jeong Hyeon Hwang, Hyun Jin Kim, Nara Shin, Sang-Ho Lee, Seung-Oh Seo, Shashi Kant Bhatia, Yung-Hun Yang

**Affiliations:** 1 Department of Biological Engineering, College of Engineering, Konkuk University, Seoul 05029, Republic of Korea; 2 Department of Pharmacy, College of Pharmacy, Jeju National University, Jeju-si 63243, Republic of Korea; 3 Department of Food Science and Technology, Seoul National University of Science and Technology, Seoul 01811, Republic of Korea

**Keywords:** Xylose regulator, polyhydroxybutyrate, lignocellulosic biomass, *Escherichia coli*, glucose, xylose

## Abstract

Polyhydroxybutyrate (PHB) production from lignocellulosic biomass is economically beneficial. Because lignocellulosic biomass is a mixture rich in glucose and xylose, *Escherichia coli*, which prefers glucose, needs to overcome glucose repression for efficient biosugar use. To avoid glucose repression, here, we overexpressed a xylose regulator (*xylR*) in an *E. coli* strain expressing *bktB*, *phaB*, and *phaC* from *Cupriavidus necator* and evaluated the effect of *xylR* on PHB production. *XylR* overexpression increased xylose consumption from 0% to 46.53% and produced 4.45-fold more PHB than the control strain without *xylR* in a 1% sugar mixture of glucose and xylose (1:1). When the *xylR*-overexpressed strain was applied to sugars from lignocellulosic biomass, cell growth and PHB production of the strain showed a 4.7-fold increase from the control strain, yielding 2.58 ± 0.02 g/l PHB and 4.43 ± 0.28 g/l dry cell weight in a 1% hydrolysate mixture. *XylR* overexpression increased the expression of xylose operon genes by up to 1.7-fold. Moreover, the effect of *xylR* was substantially different in various *E. coli* strains. Overall, the results showed the effect of *xylR* overexpression on PHB production in a non-native PHB producer and the possible application of *xylR* for xylose utilization in *E. coli*.

## Introduction

As a bio-based, bio-degradable polymer, polyhydroxybutyrate (PHB) is a promising alternative to petrochemical-based plastics [1]. PHB has potential applications in many areas, including the medical materials and packaging industry, which therefore necessitates reducing production costs to expand the coverage and sustainability of PHB bioprocessing [2-4]. One strategy for reducing PHB production cost was to lower the price of raw materials by utilizing lignocellulosic biomass as a carbon source for PHB-producing strains [5]. Lignocellulosic biomass is a highly abundant, inexpensive by-product of the agriculture and wood industry, and does not affect the human food industry, which makes it a promising substrate for PHB production [6]. However, lignocellulosic biomass becomes a sugar mixture rich in glucose and xylose when pretreated and hydrolyzed; therefore, it is necessary to overcome carbon catabolite repression (CCR) to utilize it as a carbon source for PHB-producing strains [7]. For *Escherichia coli*, glucose is preferred as a carbon source over xylose. As a result, the bacteria only utilize glucose until it is completely depleted, while xylose remains unused [8]. This phenomenon occurs because when glucose enters the cell of *E. coli*, dephosphorylated phosphotransferase protein (EIIA) is produced in the phosphotransferase system, which then acts to exclude and prevent utilization of secondary metabolic sugars. Dephosphorylated EIIA enzyme binds to an allosteric site of the promoter and prevents formation of cAMP, which is necessary for utilizing other sugars [9, 10].

As the xylose operon in bacteria has *xylAB* (xylose metabolism), *xylFGH* (xylose transporter), and *xylR* (xylose regulator) [11], many researchers have applied genetic engineering and overexpressed these genes to facilitate xylose utilization. Moreover, *xylR* in particular is one of the most commonly used genes for growing *E. coli* in xylose or mixed sugars, while overexpressed *xylR* or introduction of an *xylR* mutant to promote xylose consumption or simultaneous consumption of mixed sugars have been reported to have a positive effect on xylose consumption [12-15]. Therefore, bacterial growth could be enhanced with xylose or mixed sugars, such as glucose/xylose or xylose/arabinose, to some extent. Previous studies have already shown that the latency at transition from glucose to xylose is controlled by intracellular *xylR* availability [15]. In addition, it was reported that *xylR* overexpression in *E. coli* enabled its exponential growth in xylose and increased production of biochemicals, such as isobutanol and ethanol, in glucose/xylose mixture sugars [16, 17]. *Burkholderia sacchari*, which is an industrially viable strain for the production of xylitol, xylonic acid, and PHB, was engineered with xylose-related genes that resulted in a considerable increase in growth and PHB production via *xylR* overexpression [13]. Interestingly, despite these efforts, studies on PHB production via *xylR* overexpression in *E. coli* have not been conducted yet. As PHB is accumulated inside the cells, the impact of mixed sugar seems to be different from that of secreted products such as ethanol and isobutanol in *E. coli*, while a limitation of space required to produce PHB in a non-native PHB producer would have a different effect from that seen in native PHB-producing bacteria. In addition, considering that the cost of PHB production is highly dependent on the cost of substrate, inexpensive and abundant starting materials, such as lignocellulosic biomass, could improve the overall economy of PHB production, and feasibility testing of PHB production in *E. coli* with overexpressed *xylR* could be very helpful in further applications. Although the pretreatment cost of lignocellulosic biomass is still an obstacle, as part of the biorefinery process that breaks down biomass into several valuable products and makes the most of them, improving PHB production by increasing consumption of xylose could contribute to improving the overall economic efficiency of the process and reducing surplus resources. To confirm this, we examined the effect of *xylR* on PHB production in *E. coli* by introducing a PHB production vector, pLW487 [18-20], into *E. coli* and overexpressing *xylR*.

## Materials and Methods

### Bacterial Strains and Culture Condition

The bacteria and plasmids used in this study are listed in Table 1. *E. coli* DH5α was used for gene cloning for PHB production and xylose metabolism. The plasmid was extracted using Exprep Plasmid SV Kit (GeneAll, Korea). In addition, the extracted plasmid was inserted into various *E. coli* expression competent cells prepared in advance using the heat shock method. The cell culture was carried out in 5 ml of lysogeny broth (Difco laboratories, USA), and spectinomycin and kanamycin were selectively used for pLW487 and pRSFDuet-1. For PHA production, *E. coli* strains were cultured in 5 ml M9 minimal medium containing a 1% (w/v) glucose and xylose mixture, 0.1%(w/v) yeast extract, and 0.1 mM (w/v) isopropyl β-d-1-thiogalactopyranoside (IPTG) for 72 h at 30°C.

### RT-qPCR Analysis

For comparing the transcription level of genes related to xylose metabolism, *E. coli* HJ010 and HJ020 were cultured in 5 ml M9 minimal media containing 1% (w/v) sugar mixture (0.5% glucose + 0.5% xylose), 0.1% (w/v) yeast extract, and 0.1mM (w/v) IPTG for 72 h at 30°C. The mRNA was extracted using the RNeasy Mini Kit (Qiagen, Germany) as set forth in the manual. For cDNA synthesis, the SuperScript III First-Strand Synthesis System (Invitrogen, USA) was used. After equalizing the concentration of the synthesized cDNA equally, Real-time PCR was performed with StepOnePlus (Applied Biosystems, USA) using TOPreal qPCR 2X PreMIX (SYBR Green with high ROX, Enzynomics, Korea).

### Analysis of Dry Cell Weight and PHB Production

PHB production was determined using gas chromatography and other methods as described in previous studies, with slight modifications [21-23]. For analysis, culture samples were centrifuged at 10,000 ×*g* for 20 min, washed with deionized water twice, and suspended in 1 ml water. The suspended sample was transferred to weighted Teflon-stopped glass vials, and after lyophilization, the weight of the glass vials was re-measured to confirm the dry cell weight (DCW). For methanolysis of PHB samples, a mixture of 1 ml chloroform and 1 ml of methanol/sulfuric acid (85:15 v/v) was added to the vials and then incubated at 100°C for 2 h. The samples were then cooled in a refrigerator for 20 min to prevent the evaporation of chloroform. After adding 1 ml water, samples were mixed twice by vortexing, 10 s apart. The organic phase (bottom) was extracted using a pipette and then moved to a tube containing anhydrous sodium sulfate (Na_2_SO_4_) to remove residual moisture. Filtered samples were then injected into a GC system (Young Lin Tech., Korea) using a DB-Wax column (30 m × 0.32 mm × 0.5 μm; Agilent Technologies, USA). The split ratio was 1:10. Helium was used as the carrier gas, with a flow rate of 3.0 ml/min. A 2 μl portion of the organic phase was injected using an autosampler. The inlet was maintained at 210°C. The column oven was held at 80°C for 5 min, heated to 220°C at 20°C/min, and then held at 220°C for 5 min. Peak detection was performed using a flame ionization detector, which was maintained at 230°C.

### Lignocellulosic Biomass and Residual Sugar Analysis

The lignocellulosic biomass hydrolysate used in this study is a saccharomate® derived from *Miscanthus* and pine tree purchased from Sugaren Co., Ltd. (Korea).

For analysis of glucose and xylose, culture samples were centrifuged at 10,000 ×*g* for 10 min, and 1 ml of supernatant was filtered using a 13 mm syringe filter with 0.22 μm PVDF membrane (Chromdisc, Korea).

High-performance liquid chromatography on a PerkinElmer system equipped with a refractive index detector (Waltham, USA) and an Aminex HPX-87H column (300 × 7.8 mm internal diameter) was used to analyze the concentration of various carbon sources. A mobile phase of 0.008 N H_2_SO_4_ at a flow rate of 0.6 ml/min was used and the oven temperature was constantly maintained at 60°C during the operation.

### SDS-PAGE for Comparing the Amount of Xylose Regulator

To compare XylR amounts between various *E. coli* strains, six *E. coli* strains were cultured under optimized conditions (Fig. 2) in this experiment. Thereafter, 1 ml of the culture was divided, and the number of cells was adjusted equally to ensure that the optical density was 7.0–8.0 at 600 nm. Cell pellets were obtained via centrifugation at 15,628 ×*g* for 5 min and protein was extracted using Bugbuster. It was then mixed with 5X SDS-loading buffer and heated at 99°C for 5 min using a PCR machine (HushRun™, Biofact, Korea). The sample was loaded into the pre-prepared gel and SDS-PAGE was performed at 120 V for 2 h.

### Transmission Electron Microscopy

To prefix the cell specimens, 1 ml of each culture sample was collected, centrifuged, and mixed with Karnovsky's fixative solution containing 2% formaldehyde. The samples were then fixed with 1% osmium tetroxide in 0.05 M sodium cacodylate buffer and dehydrated in ethanol gradually (v/v) (30, 50, 70, 80, 90, and 100%). The transition step was performed using propylene. The samples were settled in different ratios of propylene oxide and Spurr's resin (v/v) (1:1 and 1:2); 100% Spurr's resin at 70°C in a dry oven was used to solidify the samples for ultramicrotome sectioning. The specimens were placed on a grid and observed under a transmission electron microscopy (TEM), with an accelerating voltage of 80 kV. TEM images of the cells and PHB granules were obtained at ×15k, ×25k, and ×40k magnification.

## Results and Discussion

### PHB Production at Different Glucose and Xylose Ratios

**Fig. 1:**
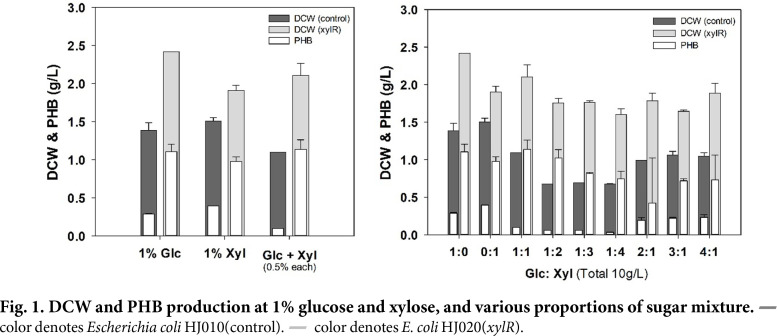
Fig. 1

As *xylR* is one of the most commonly used genes to overcome carbon catabolite repression (CCR), we aimed to validate the effectiveness of *xylR* on the production of PHB in mixed sugars using *E. coli*. To achieve this goal, we constructed HJ020 strain by introducing the PHB production vector pLW487 and the *xylR* overexpression vector pHJ02 in *E. coli* BL21 (DE3) strain. The pLW487 vector has *bktB*, *phaC*, and *phaB* genes inserted instead of the *phaCAB* operon of *Curpriavidus necator*, considering its extensibility to poly(3-hydroxybutyrate-*co*-3-hydroxyvalerate) as well as polyhydroxybutyrate (PHB). *E. coli* HJ010 strain with pLW487 and pRSFDuet-1 was used as the control strain. To see the effect of *xylR* overexpression in glucose and xylose single and mixed sugars contained in lignocellulosic biomass, the DCW and PHB accumulation of HJ010 and HJ020 were confirmed at various ratios of glucose and xylose in M9 minimal media with 0.1% (w/v) yeast extract. When glucose was used as a single carbon source, the DCW (2.42 g/l) and PHB (1.11 ± 0.01 g/l) of HJ020 were greater than the DCW (1.39 ± 0.07 g/l) and PHB (0.28 ± 0.01 g/l) of the control strain (Fig. 1). When xylose was used as a single carbon source, it was confirmed that the DCW of HJ020 strain was 1.91 ± 0.05 g/l and PHB yield was 0.98 ± 0.04 g/l, which increased compared to that seen in the control strain: DCW, 1.51 ± 0.03 g/l, and PHB, 0.40 g/l.

In contrast, the highest PHB accumulation (1.14 ± 0.09 g/l) was achieved for HJ020 when incubated in a 1:1 mixture of glucose and xylose, with a DCW of 2.11 ± 0.11 g/l. The higher the ratio of xylose at 1% of the total carbon source concentration, the lower the cell growth and PHB accumulation in both control strain and HJ020, suggesting that *E. coli* has a higher preference for glucose, even if *xylR* is overexpressed. However, the reasons for high growth and PHB accumulation in the 1:1 ratio of glucose and xylose are unclear. We could only interpret this as a result of the increased xylose consumption having a positive effect, since we could see that the xylose consumption was clearly increased (Fig. 3D). PHB production in *E. coli* requires acetyl-CoA as a precursor and NADPH as a coenzyme. Therefore, for the PHB accumulation of *E. coli*, the use of glucose as a pentose phosphate pathway is recommended rather than glycolysis. In addition, since xylose can expand the pool of the non-oxidative pentose phosphate pathway, xylose has an advantage in that it can increase the consumption of glucose to the oxidative pentose phosphate pathway. We conducted the following experiment on a 1:1 glucose-xylose mixture with the highest PHB accumulation.

### Optimization of Sugar, Nitrogen and IPTG Concentration

*E. coli* HJ020 has been genetically engineered to allow PHB accumulation, minimizing catabolite repression in lignocellulosic biomass, which primarily contains glucose and xylose. We optimized the concentration of carbon sources, nitrogen sources and IPTG to maximize PHB accumulation before introducing HJ020 strain into the lignocellulosic biomass.

**Fig. 2:**
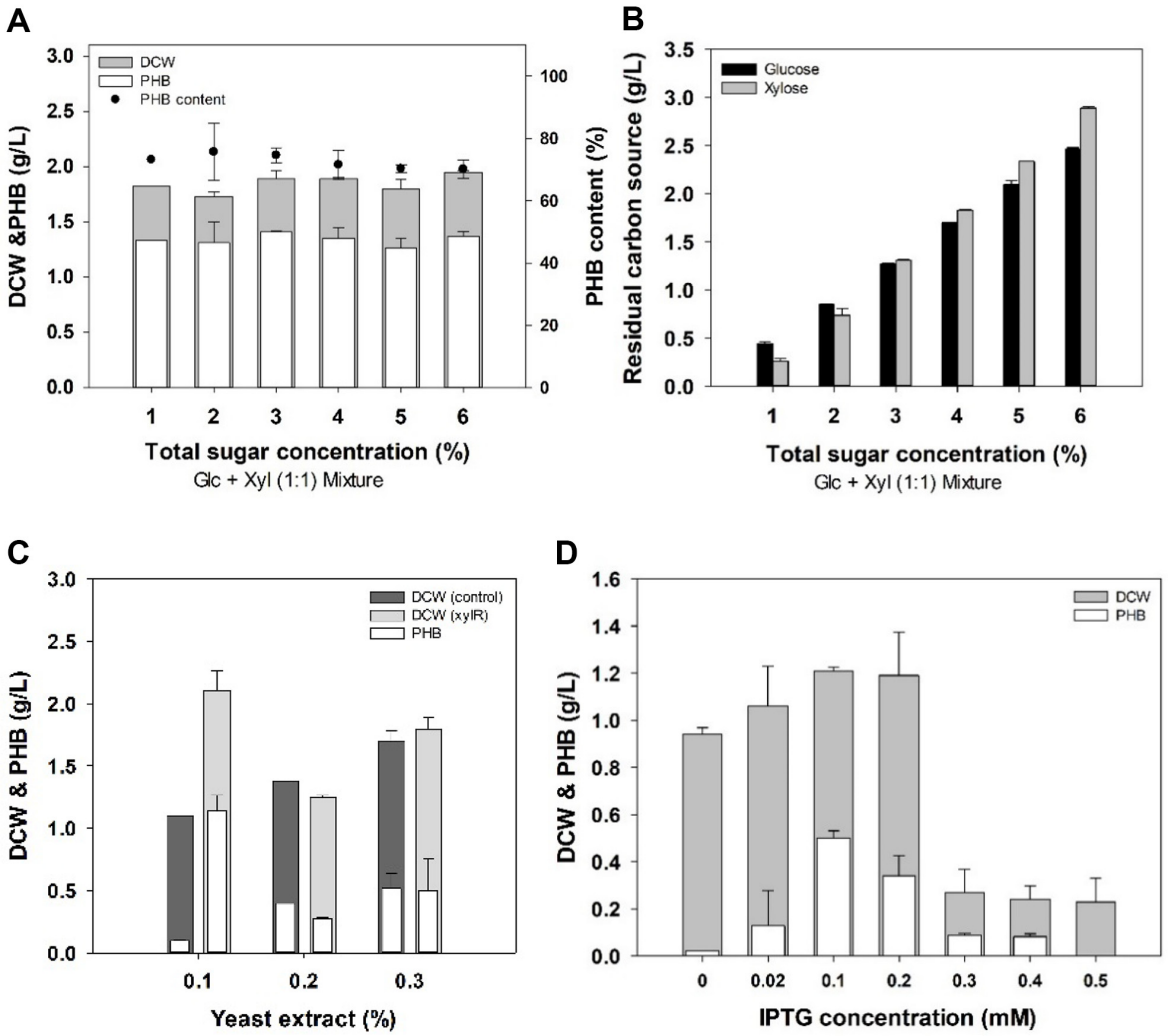
Optimization of (A) total sugar concentration (1-6%) (1:1 glucose and xylose mixture) with (B) residual glucose and xylose, (C) yeast extract (0.1-0.3%), and (D) IPTG concentration (0–0.5 mM).

First, DCW and PHB accumulation of HJ020 were confirmed at concentrations of 1–6% of glucose and xylose mixture (1:1). At 1% sugar mixture, the DCW of HJ020 was 1.82 g/l, and PHB yield (1.33 g/l) of 73.22% of the total cell weight was obtained. There was no marked change in cell growth and PHB production even though the concentration of carbon sources increased (2~6%). As shown in the Fig. 2B, *E. coli* HJ020 strain did not consume more sugar even with an increased concentration of carbon.

For optimization of the nitrogen source concentration, the cell growth and PHB production of *E. coli* HJ020 were confirmed at a concentration of 0.1 to 0.3% of yeast extract, a nitrogen source primarily used in *E. coli* culture. The optimal yeast extract concentration was 0.1%, at which time HJ020 accumulated 53.88% of PHB (1.14 ± 0.09 g/l) relative to the total cell weight (2.11 ± 0.11 g/l).

IPTG concentration was optimized at 1% sugar mixture and 0.1% yeast extract to control the optimal expression of *xylR* expressed under the T7 promoter. As shown in the Fig. 2D, it was confirmed that HJ020 produced the highest DCW (1.21 ± 0.01 g/l) and PHB (0.50 ± 0.02 g/l) at 0.1mM IPTG concentration.

### Time-Dependent Monitoring of PHB Production and Sugar Utilization

**Fig. 3:**
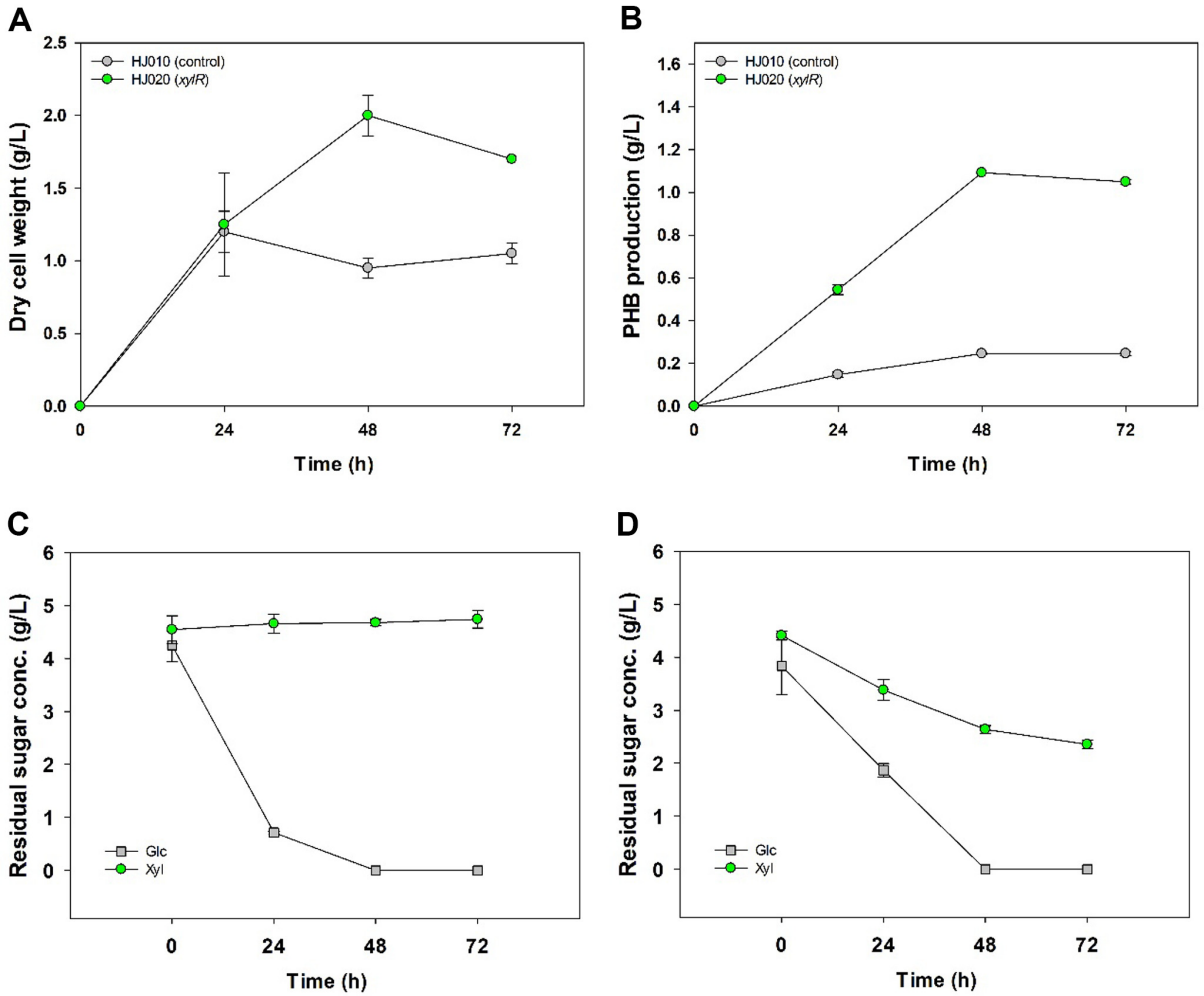
Time-dependent (A) dry cell weight (DCW), (B) PHB production and sugar utilization of (C) *E. coli* HJ010 (control) and (D) *E. coli* HJ020 (*xylR*) in optimized condition.

**Fig. 4:**
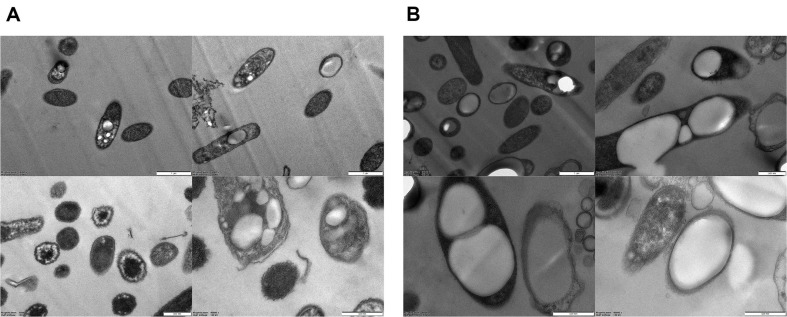
Transmission electron microscopy (TEM) analysis of the (A) *E. coli* HJ010 (control) and (B) HJ020 (*xylR*); (×15k, ×25k ×40k).

Under optimized conditions, time-dependent cell growth, PHB accumulation, and sugar mixture consumption of *E. coli* HJ010 and HJ020 were confirmed. For HJ010 (control), the highest DCW (1.2 ± 0.1 g/l) was obtained by quickly consuming only glucose for 24 h after incubation initiation. In addition, all remaining glucose was consumed between 24 and 48 h, and the highest PHB production (0.25 ± 0.00 g/l) was confirmed at 48 h (Fig. 3). Considering that *E. coli* DH5a and BL21 (DE3) strains consumed all glucose within 24 h under the same culture conditions (Fig. S1), it is possible that the accumulation of PHB in cells due to the introduction of vector reduced the rate of glucose consumption in *E. coli*. On the other hand, in the case of *xylR*-overexpressing *E. coli* HJ020 strain, the highest DCW (2.0 ± 0.1 g/l) and PHB (1.09 g/l) production were confirmed at 48 h, and in the case of PHB, it was 4.45-fold higher than HJ010 (Figs. 3A and 3B). As a result of analyzing the residual sugar consumption in HJ020, we interpret the enhanced cell growth and increased PHB production as a direct outcome of the efficient utilization of xylose attributed to the overexpression of *xylR*. In contrast to HJ010 strain, which did not consume xylose, HJ020 strain was shown to have consumed 46.53 ± 0.58% of xylose out of 5 g/l of xylose (Fig. 3D). Because of *xylR* overexpression, the consumption rate of glucose decreased slightly compared to control. The decrease in the consumption rate of glucose due to this *xylR* overexpression is similar to previous studies in which *xylR* mutant introduction increased xylose consumption and reduced glucose consumption [17]. As a result, the overexpression of *xylR* could have a positive effect on the PHB accumulation of *E. coli*, as the rate of consumption of glucose decreased slightly, although the total consumption of mixed sugars increased at 72 h. Further, we also confirmed the morphology of HJ010 and HJ020 at 72 h by using transmission electron microscopy (TEM) (Fig. 4). As a result, HJ020 strain overexpressed *xylR* accumulated more PHB in the cell and generally showed a larger cell size than the control strain.

### PHB Production with *xylR*-Overexpressing Strain by Using Lignocellulosic Biomass Hydrolysate

**Fig. 5:**
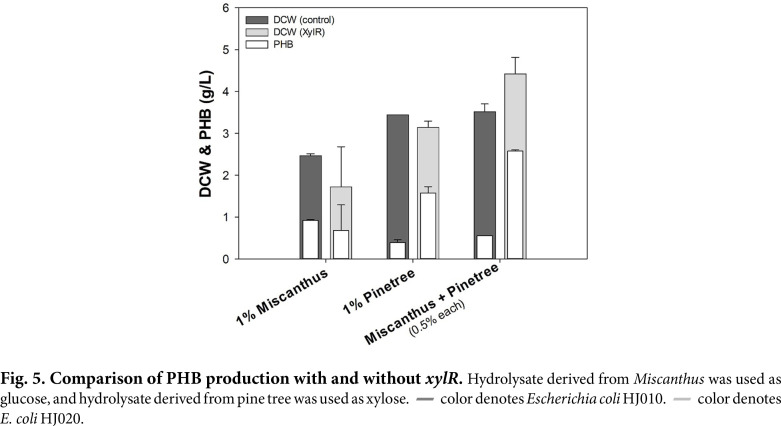
Fig. 5

To confirm the effect of *xylR* overexpression in the actual lignocellulosic biomass, PHB production and cell growth were confirmed from lignocellulosic biomass hydrolysate using HJ020. *Miscanthus*-derived biosugar contained a high proportion of glucose showing 113:4 ratio to xylose, and pine tree-derived biosugar contained a high proportion of xylose, showing a 36:103 ratio for glucose to xylose (Table S1). As shown in Fig. 5, HJ020 showed lesser PHB production than control with hydrolysate of *Miscanthus* because of the extremely high ratio of glucose to xylose. In the case of pine tree, HJ020 accumulated 4-times more PHB (1.57 ± 0.11 g/l with 50.08 ± 4.97% of PHB content) than control at 1% pine tree biosugar, and there was no significant difference in cell growth. The maximum PHB concentration and cell growth were achieved in the *Miscanthus* + pine tree sugar mixture, with a 74:53 ratio of glucose to xylose. HJ020 increased PHB yield (2.58 ± 0.02 g/l with 58.44 ± 3.21% of PHB content) by 4.7 times compared to that seen in the control, and the DCW (4.43 ± 0.28 g/l) further increased by 1.6 times. Although *xylR* overexpression was effective in high-xylose conditions, it seemed to be the best with a similar ratio of glucose and xylose. On the other hand, cell growth and PHB production when using lignocellulosic biomass hydrolysate were generally higher than when using glucose and xylose. This was contrary to our expectation that cell growth would decrease because the lignocellulosic biomass hydrolysate contains inhibitors, such as furfural and acetic acid. The lignocellulosic hydrolysate contains a variety of amino acids, peptides, metal ions, and aromatics, as well as carbohydrates, which were expected to accumulate high DCW and PHB by supplementing trace elements lacking only with the use of yeast extract [24-27].

### Analysis of Xylose Operon Transcription Level with *xylR* Introduction

**Fig. 6:**
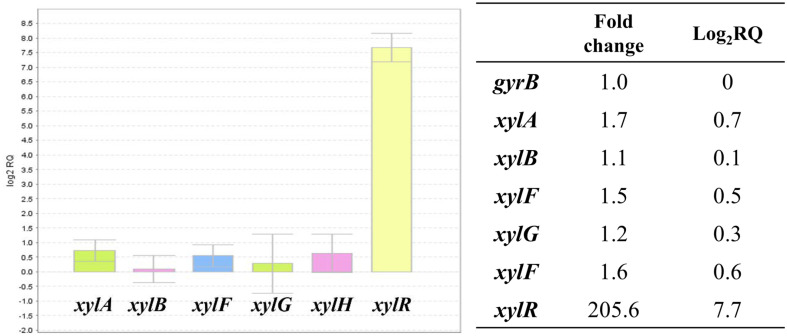
The mRNA expression of *xylAB* and *xylFGH* operons because of *xylR* overexpression was observed using RT-qPCR.

To evaluate the transcription level of *xylR* and the resulting expression of xylose operon, *E. coli* HJ020 strain was cultured in M9 minimal medium with 1% sugar mixture for 72 h, and then mRNA was extracted and RT-qPCR was performed. *E. coli* HJ010 strain was used as control. The overexpressed *xylR* under the T7 promoter was 205.6- fold more expressed than the control. In HJ020, a strain overexpressing *xylR*, expression of *xylA* (1.7-fold), *xylB* (1.1-fold), *xylF* (1.5-fold), *xylG* (1.2-fold) and *xylH*(1.6-fold), which are genes belonging to xylose operon, further increased (Fig. 6). As expected, *xylR* overexpressed in the *E. coli* HJ020 strain positively controlled *xylAB* and *xylFGH* operon.

### PHB Production in Different *E. coli* Strains

To verify if the overexpression of *xylR* could universally enhance PHB production in mixed sugars of glucose and xylose, BL21 (DE3) (HJ020) strain and five other *E. coli* expression strains (C41 (DE3), C43 (DE3), JM109 (DE3), HR200 (DE3), and KSYH (DE3)) were introduced with pLW487 and pHJ02 (*xylR*). The culture was conducted under optimized conditions in the previous experiment, and PHB and DCW were measured before and after *xylR* introduction.

**Fig. 7:**
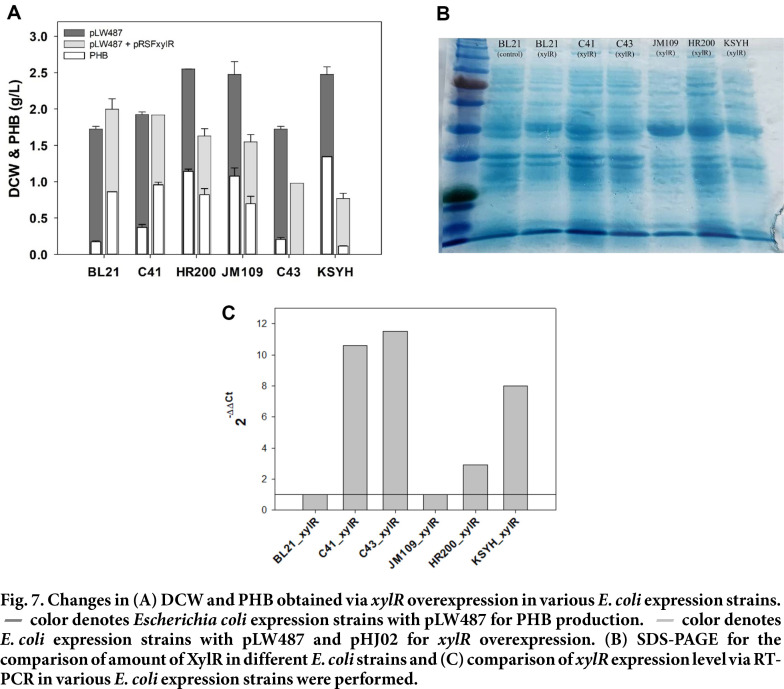
Fig. 7

As expected, BL21 (DE3) showed increased PHB production by overexpression of *xylR*. In the case of *E. coli* C41 (DE3), there were no significant differences observed in terms of DCW when *xylR* was overexpressed. PHB production was increased by 4 times in BL21 and 1.6 times in C41, accumulating PHB of 0.86 g/l and 0.96 ± 0.03 g/l, respectively (Fig. 7A). This suggested that *xylR* overexpression in *E. coli* had a positive effect on PHB production with the mixed sugar. However, overexpression of *xylR* did not have a positive effect on all *E. coli* strains. When *xylR* was overexpressed in C43 (DE3), JM109 (DE3), KSYH (DE3), and HR200 (DE3) strains, both the DCW and PHB accumulation were shown to be decreased. This type of a result was reported in *Burkholderia sacchari*. When *xylR* expression was induced with low-level IPTG (6.25 μM), a 66% increase in cell growth was achieved, and high-level IPTG induction (50 or 500 μM) was reported to delay growth [13]. In addition, in the case of *E. coli* MG1655 strain, it has been reported that high expression levels of *xylR* in arabinose and xylose mixed sugars caused growth delay [12]. As stated above, the high expression level of *xylR* may have negatively affected growth, and we hypothesized that the four strains with reduced growth would have expressed higher amounts of *xylR* at the same IPTG concentration. To prove this hypothesis, SDS-PAGE and RT-PCR were attempted to compare *XylR* protein amounts and *xylR* expression levels for various *E. coli* strains (Figs. 7B and 7C). However, there was no significant correlation between the experimental results and the decline in growth in *E. coli* strains. Even the comparison between BL21 (DE3) and C41 (DE3), which showed increased PHB production, the correlation was hard to detect. Moreover, strains with decreased PHB production showed quite different patterns of protein and mRNA expression. As a result of confirming the expression of *xylR* through RT-PCR and SDS-PAGE, the expressions of mRNA and protein between strains were not proportional. In the case of C41, C43, and KSYH strains, mRNA expression was high, but protein expression was relatively low. On the other hand, in the case of JM109 and HR200, the expression of mRNA was relatively low, but the expression of protein was high. The effect of *xylR* in one strain can be associated with the expression of *xylR*, but the effect of *xylR* on multiple strains can be difficult to relate to the expression. This relationship between *xylR* overexpression and decrease in growth may have more complex causes than reported and expected. Considering various *E. coli* strains had many manipulations with sugar metabolisms, the interaction of sugar metabolisms seemed to affect xylose utilization and PHB production combined with *xylR*.

## Conclusion

We found that *E. coli* HJ020, an *xylR*-overexpression strain with pLW487 inserted for PHB production, is effective in PHB production in a 1% mixture of glucose and xylose (1:1). Under optimized conditions, the DCW (2. 0 ± 0.1) of HJ020 was 1.7-fold higher than the control strain and produced 4.45-fold more PHB (1.09 ± 0.00). Furthermore, the *xylR* overexpression of HJ020 was effective in lignocellulosic biomass hydrolysate rich in glucose and xylose. This strain produced 2.58 ± 0.02 g/l of PHB in a 1% *Miscanthus* and pine tree mixture and accumulated 4.7-fold more PHB than the control strain. We confirmed that these results were due to the effective activation of xylose operon by overexpressed *xylR*. *xylR* overexpression can be presented as a strategy to increase PHB production using *E. coli* BL21 from lignocellulosic biomass.

To verify the universality of *xylR* overexpression, we tested its effects on the production of PHB in different *E. coli* expression strains. However, among the six strains, only BL21 and C41 strains showed an increase in PHB production under *xylR* overexpression, and in the remaining four strains (C43, JM109, HR200 and KSYH), *xylR* overexpression resulted in a decrease in DCW and PHB production. Although the positive effects of *xylR* in BL21 are apparent, the control of CCR might be more complex and strain-dependent than what has been explored in this study.

## Supplemental Materials

Supplementary data for this paper are available on-line only at http://jmb.or.kr.



## Figures and Tables

**Table 1:** Bacterial strains and plasmids used in this study.

Strain or plasmid	Description	Reference
Bacterial strains		
*E. coli* strains		
DH5α	F^-^ φ80*lacZ* M15 *endA* *recA* *hsdR*(rk^-^ mk^-^) *supE thi gyrA relA* Δ(*lacZYA-argF*) U169	Laboratory stock
BL21(DE3)	F^-^ *ompT hsdS_B_*(r_b_^-^m_b_^-^) *gal dcm*	Novagen
C41(DE3)	F^-^ *ompT hsdS_B_*(r_b_^-^m_b_^-^) *gal dcm*, BL21(DE3) derivative	[28]
C43(DE3)	F^-^ *ompT hsdS_B_*(r_b_^-^m_b_^-^) *gal dcm*, C41(DE3) derivative	[28]
HR200(DE3)	Δ*pflb*, Δ*ldhA*, Δ*adhE*, Δ*fnr*, containing pACYC::*pntAB*, BW25113(DE3) derivative	[29]
JM109(DE3)	recA1, endA1, gyrA96, thi, hsdR17, supE44, relA1, λ-, Δ(lac-proAB), [F' traD36, proAB, lacI^q^ laqZΔ M15]	[30]
KSYH(DE3)	ΔaraBAD, ΔrhaBAD, BW25113(DE3) derivative	[31]
HJ010	BL21(DE3) containing pLW487 and pRSFDuet-1	This study
HJ020	BL21(DE3) containing pLW487 and pHJ02	This study
Plasmids		
pLW487	Spec^R^ pEP2-based, plasmid carrying *bktB*, *phaB* and *phaC* under trc promoter from *R.eutropha*	[32]
pRSFDuet-1	Km^R^, RSF ori	[16]
pHJ02	Km^R^, pRSFDuet-1-based plasmid carrying *xylR* from *Escherichia coli* under T7 promoter	[16]

**Table 2:** PHB production of different microbes genetically modified to increase PHB production by facilitating xylose consumption.

Strain	Carbon sources	Culture mode	Strategy	CDW (g/l)	Contents (%)	PHB (g/l)	Ref
*E. coli*							
JM109(DE3)	glucose + xylose	Fed-batch	Δ*ndh*, Overexpression of *xylAB_Bs_* and *araE_Bs_*	53.2	-	21.0	[33]
K12(DE3)	xylose	Flask	Δ*rpe*, Δ*poxB*, Δ*ackA*, Overexpression of *xfp_Lr_*, *pta_Ec_* and *gapC_Ca_*	6.57 ± 0.17	12.83 ± 1.14	0.84 ± 0.07	[34]
BL21(DE3)	*Miscanthus* + Pinetree hydrolysate	Test tube	Overexpression of *xylR*	4.43 ± 0.28	58.44 ± 3.21	2.58 ± 0.02	This study
*C. necator*							
NCIMB11599	sunflower stalk hydrolysate	Flask	Overexpression of *xylAB_Ec_*		72.53	7.86	[35]
	glucose + xylose	Test tube	Co-culture with *Bacillus* sp. SM01	5.71 ± 0.03		3.55 ± 0.02	[36]
*B. sacchari*							
	xylose	Batch	*xylR* overexpression	8.02 ± 0.44	71.07 ± 2.46	5.7	[13]
	xylose	Fed-batch	*xylAB* overexpression	16.89	67	11.29	[37]
	xylose + hexanoate	Fed-batch	Overexpression of *tktB_Bsa_*	20.2-23.8	-	9.1-12.1	[38]
*H. bluphagenesis*							
	xylose	Batch	Overexpression of *HEO0208*, *xylA* and *xfp*	8.81 ± 0.36	61.04 ± 1.04	5.37 ± 0.13	[39]

Abbrebiations: *E. coli*, Ec, *Escherichia coli*; Bs, *Bacillus subtilis* 168; Lr, *Lactobacillus rhamnosus* CGMCC1.120; Ca, *Clostridium acetobutylicum* ATCC 824; *C. necator*, *Cupriavidus necator*; *B. sacchari*, Bsa, *Burkholderia sacchari*; *H. bluphagenesis*, *Halomonas bluphagenesis*
