# Supplementary material for: Validating a Xylose Regulator to Increase Polyhydroxybutyrate Production for Utilizing Mixed Sugars from Lignocellulosic Biomass Using Escherichia coli
Source: J Microbiol Biotechnol. 2023 Sep 18;34(3):700–9. doi: 10.4014/jmb.2306.06006 (PMC11016755; doi:10.4014/jmb.2306.06006)

J. Microbiol. Biotechnol.
https://doi.org/10.4014/jmb.2306.06006

Supplementary Table and Figure

Supplementary Table S1. Composition of the biomass hydrolysate used in this study.

Biomass	Composition		Concentration (g/L)
Miscanthus	Monosugar	Glucose	112.63±0.078
		Xylose	3.677±0.038
	Byproduct	Acetic acid	0.018±0.004
Pinetree	Monosugar	Glucose	35.527±0.01
		Xylose	102.560±0.023
		Mannose	21.973
		Galactose	14.97
		Arabinose	6.05
	Byproduct	Formic acid	0.928±0.001
		Acetic acid	0.107
		Furfural	0.056

Supplementary Figure S1. DCW and Residual sugar consumption of DH5α and BL21 in optimized conditions.

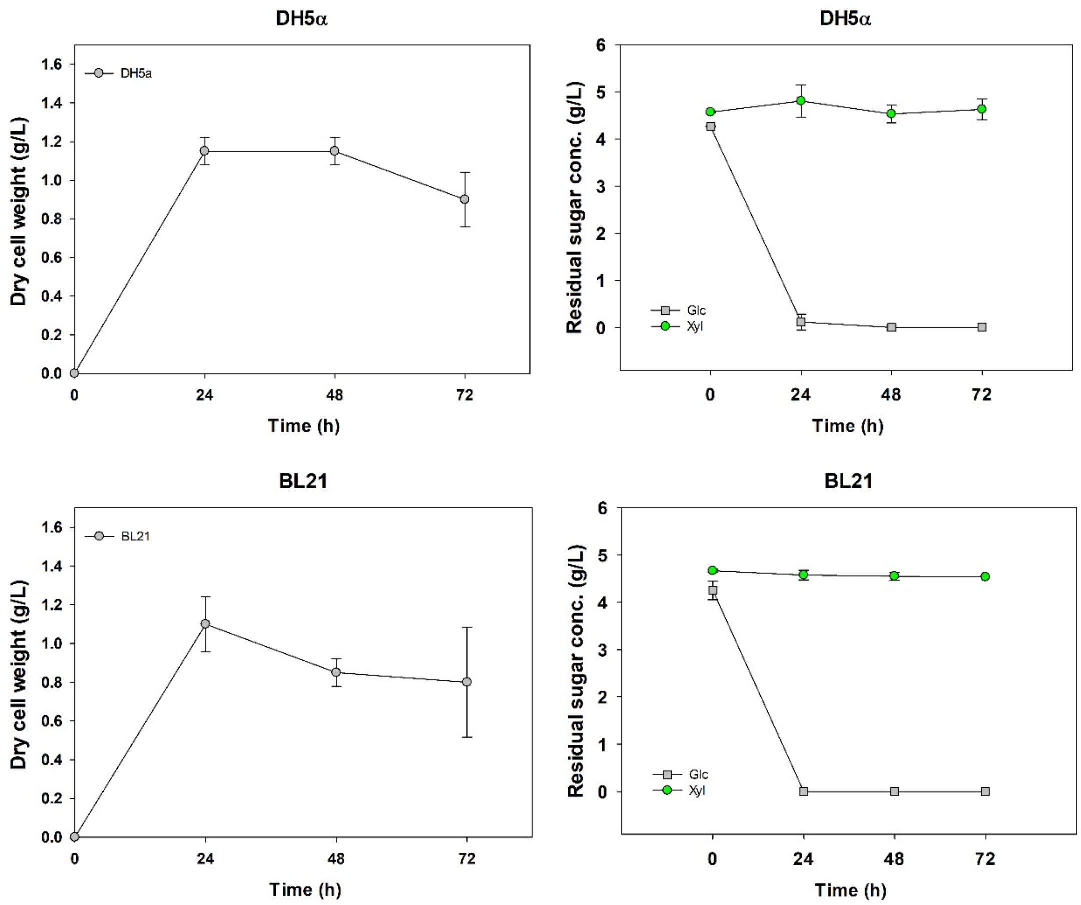

Supplement: Supplementary file 1 [file jmb-34-3-700-supple.pdf]
